# Aquaporin 5 and Nuclear Factor Erythroid 2-Related Factor 2 Upregulation in Tumor and Stroma Is Associated with Poor Pathological Complete Response to Neoadjuvant Chemotherapy in Breast Cancer

**DOI:** 10.1159/mpp/adpag003

**Published:** 2026-08-10

**Authors:** Ilija Guteša, Sanda Bubanović, Tonči Vodopić, Monika Mlinarić, Krešimir Grgat, Ana Čipak Gašparović

**Affiliations:** a University Hospital for Tumors, University Hospital Centre “Sestre milosrdnice”, Zagreb, Croatia; b Institute for Pathology, Cantonal Hospital Baden, Baden, Switzerland; c General Hospital Dubrovnik, Dubrovnik, Croatia; d Ruđer Bošković Institute, Zagreb, Croatia

**Keywords:** Aquaporin 3, Aquaporin 5, Nuclear factor erythroid 2-related factor 2, Immunohistochemistry, Triple-negative breast cancer, HER2+ breast cancer

## Abstract

**Objective:**

Breast cancer remains a leading cause of global mortality in women, with therapy resistance posing a major challenge to successful treatment. Chemotherapy-induced oxidative stress can trigger adaptive survival mechanisms through the antioxidative transcription factor NRF2 and membrane pores known as peroxiporins. Peroxiporins regulate cellular redox homeostasis by controlling the flux of H_2_O_2_, potentially driving the development of chemoresistance in aggressive subtypes. This study investigated the expression of peroxiporins, AQP3 and AQP5, and the antioxidative transcription factor NRF2, before and after chemotherapy to determine their role in chemotherapy resistance in HER2-positive and triple-negative breast cancer.

**Subjects and Methods:**

In this retrospective study, we analyzed the expressions of AQP3, AQP5, and NRF2 by immunohistochemical staining in paired clinical biopsy samples of HER2-positive (*N* = 75) and triple-negative (*N* = 56) breast cancer patients. We evaluated target protein expression across both tumor and surrounding stroma within these tissue samples.

**Results:**

Chemotherapy significantly increased AQP5 and NRF2 expression in both tumor cells and stroma across both subtypes. However, when stratified by therapy response, significant upregulation of AQP5 and nuclear NRF2 was observed only in the pathological incomplete response group. In contrast, patients achieving a complete response showed no significant changes in protein expression post-therapy.

**Conclusion:**

Chemotherapy is associated with increased AQP5 expression and NRF2 nuclear translocation within both tumor cells and stroma. These post-therapy molecular changes are significantly associated with a lack of pathological complete response, suggesting that AQP5 and NRF2 may be involved in resistance to chemotherapy in HER2-positive and TNBC.

## Plain Language Summary

Breast cancer is a major health challenge, and the biggest obstacle that doctors face is chemoresistance, when cancer cells survive and fight the therapy. Here, we looked at the two aggressive breast cancers, HER2-positive and triple-negative types, and the new ways how they might fight back the therapy. We focused on specific proteins that help cells in fighting oxidative attack: NRF that is master switch for antioxidative defense and two peroxiporins, AQP3 and AQP5, located in cell membrane that control the flow of hydrogen peroxide, thereby controlling the stress. By comparing tumor samples taken before and after chemotherapy, the pattern emerged. In patients whose tumors did not respond well to therapy significantly increased AQP5 and NRF2, while in patients whose tumors completely disappeared after therapy did not increase these proteins. These results indicate that these two proteins help tumor in fighting therapy and could serve as flags to predict the therapy outcome.

**Highlights of the Study**

- Chemotherapy increases expression of aquaporin 5 and nuclear factor erythroid 2-related factor 2 in both tumor and stroma cells.
- Aquaporin 5 is upregulated stroma of patients with incomplete response to chemotherapy.
- Nuclear factor erythroid 2-related factor 2 localizes in tumor nuclei in patients with incomplete response to chemotherapy.

## Introduction

While progress in treatments and diagnostics has improved patient outcomes, breast cancer continues to be a public health challenge and the leading cause of cancer-related death among women in Western countries. Recent epidemiological data from Europe indicate that breast cancer has the highest annual incidence in the female population, accounting for nearly 29% of all female cancer diagnoses, and remains a leading cause of mortality in women [1]. In order to determine the specific subtype of breast cancer, immunohistochemical staining is used to determine estrogen receptor (ER), progesterone receptor (PR), human epidermal growth factor receptor 2 (HER2), and the antigen Kiel 67 (Ki67) proliferation index. High Ki67 expression indicates highly proliferative breast cancer, whereas HER2 expression additionally enhances proliferation, thereby contributing to tumor aggressiveness. Fortunately, HER2 is an effective therapeutic target; when targeted therapies are combined with standard chemotherapy, they provide high survival rates [2]. On the other hand, triple-negative breast cancer (TNBC), which lacks ER, PR, and HER2 expression, lacks an effective therapeutic target and is highly proliferative, resulting in a highly aggressive phenotype. Unless these patients are carriers of *BRCA1/2* mutations, treatment options are limited to chemotherapy and surgery [3]. Metastases and therapy resistance are the leading causes of cancer-related mortality. Therefore, understanding the molecular changes and mechanisms behind these processes is crucial for developing successful therapeutic strategies. Potential targets that have emerged include various aquaporins (AQPs). AQPs are transmembrane pores that facilitate the transport of water, glycerol, hydrogen peroxide, and other small molecules across the cell membrane [4]. In mammals, 13 different AQPs (AQP0–AQP12) are distinguished, which are grouped according to their primary substrate: orthodox AQPs that transport water (AQP0, AQP1, AQP2, AQP4, AQP5, AQP6, AQP8); aquaglyceroporins that transport glycerol (APQ3, AQP7, AQP9, and AQP10); and s-AQPs which are exclusively located inside the cell (AQP11 and AQP12) [5]. In addition, AQPs that transport hydrogen peroxide are called peroxiporins (AQP1, AQP3, AQP5, AQP8, AQP9, and AQP11) comprising members of all three initial groups [6]. AQPs contribute to numerous cellular processes and signaling pathways by controlling the flux of water, hydrogen peroxide, and glycerol. In cancer, AQPs are frequently upregulated and often correlate with poorer prognosis [3, 7]. In breast cancer, AQP3 and AQP5 have been identified as potential prognostic markers as their concurrent expression in TNBC correlates with worse outcome [8]. In vitro results point to the important role of these AQPs in (cancer) cell migration and proliferation [9, 10], as well as their role in the therapy response [11]. In vivo studies in severe combined immunodeficient mice revealed that while AQP3 knockdown does not alter primary tumor growth, it significantly impairs cell migration, suppressing metastatic potential when knocked down and enhancing it when overexpressed [12].

The spatial regulation of hydrogen peroxide is important due to its role in signaling pathways. Therefore, controlling its localization and concentration via transmembrane flux remains crucial for (cancer) cell survival. Nuclear factor erythroid 2-related factor 2 (NRF2) is the major antioxidant transcription factor that controls the expression of more than 200 genes involved in antioxidant protection, detoxification, and metabolism [13]. In breast cancer, NRF2 is highly expressed in cancer cells, while in the stroma, NRF2 expression is increased and correlates with breast cancer aggressiveness [14]. Furthermore, mutations either in *NFE2L2* (gene encoding NRF2) or in *KEAP1*, its inhibitor, lead to constitutive activation, thereby increasing the cancer cell resistance toward oxidative stress, including cancer therapy [15]. As recognized by Hanahan and Weinberg [16] in the hallmarks of cancer, the tumor surrounding microenvironment influences drug delivery and can actively support, or in some cases inhibit, tumor growth. Consequently, peroxiporins participate in small molecule flux, and antioxidant regulators (NRF2) can participate in microenvironmental remodeling and signaling. Investigating alterations of AQPs and NRF2 in the stroma could provide crucial insights into how nonmalignant supporting cells contribute to the mechanisms of therapy resistance.

Based on these observations, we hypothesized that the regulation of both AQPs (AQP3 and AQP5) and NRF2 could provide a critical survival advantage to the tumor under the therapy. Specifically, we hypothesized that elevated expression levels of these proteins, in both tumor and stroma, would correlate with the presence of residual invasive tumor after therapy (non-pathological complete response [non-pCR]). To test this, our objectives were to evaluate the expression profiles of these peroxiporins and NRF2 in paired pre- and post-therapy tumor and stromal tissue samples from patients with HER2+ and TNBC subtypes. Additionally, we aimed to define the statistical correlations and co-expression patterns between AQP3/AQP5 and NRF2 within each clinical cohort in relation to the therapeutic response.

## Subjects and Methods

### Study Design and Ethics

The retrospective study included 131 patients from the University Hospital for Tumors, University Hospital Centre “Sestre milosrdnice.” Patients were referred to the University Hospital for Tumors, University Hospital Centre “Sestre milosrdnice” with suspicion of breast cancer which was confirmed by core biopsy. Inclusion criteria required patients to have histologically confirmed, primary invasive HER2+ breast cancer or TNBC immunophenotype with no prior history of radiotherapy or systemic treatment, and available paired pre- and post-therapy tissue blocks. Baseline receptor status was determined according to ASCO/CAP guidelines; TNBC was defined as <1% ER/PR expression and HER2 negativity (IHC 0/1+ or 2+ with negative FISH), while HER2+ required an IHC score of 3+ or positive FISH amplification. Patients with stage IV metastatic disease or inflammatory breast cancer were excluded. Patients received therapy according to the core biopsy results and the cancer’s stage. All the patients received neoadjuvant chemotherapy administered intravenously according to institutional protocols. Anthracycline-based chemotherapy (Adriamycin and cyclophosphamide, AC) consisted of doxorubicin (60 mg/m^2^) and cyclophosphamide (600 mg/m^2^) administered as four cycles of dose-dense AC every 14 days with prophylactic long-acting granulocyte colony-stimulating factor support, while 3 patients received six cycles of standard AC every 21 days, depending on the treatment protocol used during the study period. Patients with TNBC subsequently received 12 weekly cycles of paclitaxel (80 mg/m^2^). Patients with HER2-positive breast cancer received 12 weekly cycles of paclitaxel combined with trastuzumab (loading dose 8 mg/kg followed by 6 mg/kg maintenance dose) and pertuzumab (loading dose 840 mg followed by 420 mg maintenance dose), according to the standard of care applicable at the time of treatment. Patients who did not complete the planned neoadjuvant treatment were excluded from the analysis.

Following completion of neoadjuvant systemic therapy, definitive breast surgery with appropriate axillary staging or surgery was performed according to institutional protocols within the timeframe recommended by contemporary international guidelines and the EUSOMA Quality Indicators. Based on the pathological findings, patients were classified as having achieved either a pathological complete response (pCR), defined as the absence of residual invasive carcinoma in the breast and regional lymph nodes (ypT0/is ypN0, allowing residual ductal carcinoma in situ [DCIS]), or non-pCR, defined as the presence of residual invasive.

### Immunohistochemical Staining for AQP3, AQP5, and NRF2

Breast cancer tissue samples collected by core biopsy and resection were dehydrated in an increasing ethanol gradient and xylene. After dehydration, samples were embedded in paraffin and cut into 5-µm thin sections for specific immunohistochemical staining. Following the rehydration through xylene and decreasing gradient of ethanol to phosphate-buffered saline, antigen retrieval was performed in citrate buffer pH 6.0 at 85°C for 35 min for all antibodies. Samples were then washed three times with TRIS-buffered saline with Tween 20 for 5 min and incubated with primary antibodies: anti-NRF2 (1:100, ab31163; Abcam, UK), anti-AQP3 (1:50, sc-518001, Santa Cruz Technology, USA), and anti-AQP5 (1:50, sc-514022, Santa Cruz Technology) overnight at room temperature. The next day, samples were washed three times in TRIS-buffered saline with Tween 20 for 5 min and incubated with secondary anti-mouse antibody (EnVision, Dako, Denmark) for NRF2, AQP3, and AQP5. The antigen-antibody complex was visualized with diaminobenzidine (Dako), yielding a brown color for the presence of the antigen. Nuclei were then counterstained with hematoxylin, yielding a blue color. Negative controls were established by omitting the primary antibodies. Intensity and localization of positivity for each antigen were analyzed by two experienced pathologists (author 2 and author 3) blinded to the clinical characteristics, treatment arms, and pathological outcomes of the patients. For all our target proteins, positivity was separately evaluated for the tumor cells and the surrounding stroma. Furthermore, for AQPs, cellular positivity was defined by staining present in the cytoplasm, the plasma membrane, or both compartments simultaneously. For NRF2, expression was further stratified into nuclear and cytoplasmic localization. Results are presented as the percentage of positive cells within each respective compartment (percentage of positive tumor cells among total tumor cells, and the percentage of positive stromal cells among total stromal cells; representative immunohistochemical staining results are shown in Fig. 1).

**Fig. 1.**
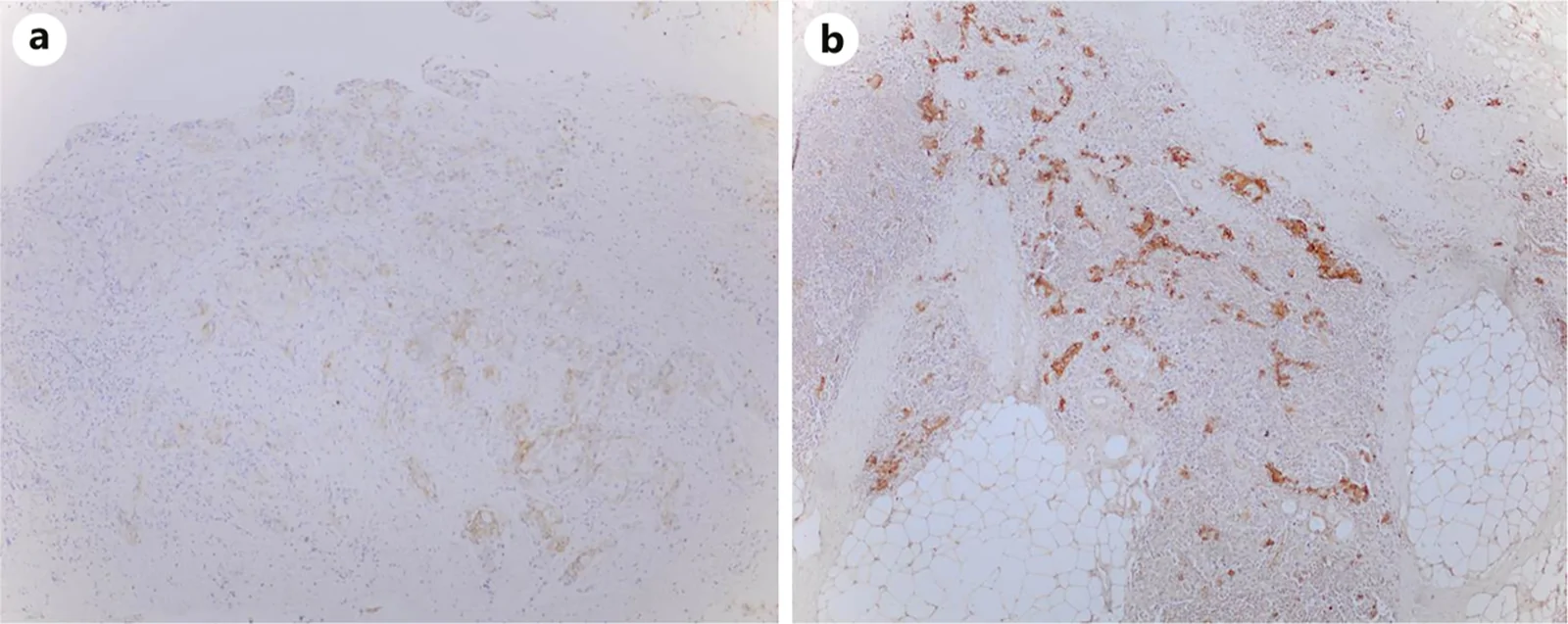
Representative immunohistochemical staining of AQPs in TNBC non-pCR patient. **a** AQP3 expression. **b** AQP5 expression; magnification ×100.

### Statistical Analyses

Results are presented as mean ± standard error, unless otherwise stated. Normal distribution was tested by the Shapiro-Wilk test, and relations between groups were tested using the χ^2^ test. Because tissue samples represent paired, repeated measures from the same patients before and after therapy, differences between the groups were tested by two-way ANOVA with Tukey’s post hoc test to account for within-subject dependencies. Correlations between the studied markers are tested by Spearman’s correlation, using intervals according to Schober et al. [17]: 0.3–0.69 for moderate correlation, 0.7–0.89 for strong correlation, and 0.9–1 for very strong correlation. Values of *p* < 0.05 are considered significantly different. Statistical analyses were performed using GraphPad Prism 8.0 (GraphPad Software, San Diego, CA, USA) or jamovi (version 2.3.28) [18].

## Results

The study included 131 patients diagnosed with breast cancer who underwent neoadjuvant chemotherapy, of whom 56 (42.7%) were diagnosed with TNBC and 75 (57.3%) with HER2+ breast cancer. Patients were sub-stratified according to therapy response: pCR (48, 36.6%) and non-pCR (83, 63.4%). In the pCR cohort, 13 (17.1%) were TNBC and 35 (72.9%) were HER2+ patients, whereas in the non-pCR cohort 43 (51.8%) were TNBC and 40 (48.2%) were HER2+ patients. The average age (57.3 years) and average tumor size (36.9 mm) showed no statistically significant differences across the immunophenotypes or by pCR/non-pCR groups (*p* = 0.130 for both). There was a statistically significant difference in baseline Ki67 expression between TNBC and HER2+ patients (*p* < 0.001), whereas no significant difference was observed regarding ultimate pCR status within each group. The detailed descriptive clinical characteristics are presented in Table 1.

**Table 1.** Descriptive statistics of patient and tumor characteristics grouped by immunophenotype and response to neoadjuvant chemotherapy

​	HER2+ (*n* = 75)		TNBC (*n* = 56)	
	non-pCR (*n* = 40)	pCR (*n* = 35)	non-pCR (*n* = 43)	pCR (*n* = 13)
Age (years)				
Mean±SD	60.9±11.3	56.2±10.1	56.3±12.2	53.1±11.3
Median (range)	62.0±11.3	57 (37–81)	56 (30–81)	54 (37–72)
Tumor size (mm)				
Mean±SD	37.8±17.2	35.6±15.7	37.9±18.1	34.2±24.9
Median (range)	34.5 (15–100)	30 (14–80)	34 (10–100)	27 (12–97)
Ki67 index (%)				
Mean±SD	39.4±21.6	40.5±19.8	61.3±21.2	54.2±22.8
Median (range)	37.5 (10–89)	35.0 (13–89)	60 (18–96)	59 (16–92)
RCB score				
Mean±SD	2.16±1.03	0.00±0.00	2.56±1.24	0.00±0.00
Median (range)	2.05 (0.00–4.42)	0.00 (0.00–0.00)	2.45 (0.00–4.77)	0.00 (0.00–0.00)

RBC, residual cancer burden; pCR, pathological complete response; non-pCR, non-pathological complete response; TNBC, triple-negative breast cancer; HER2+, HER2-positive breast cancer.

### Immunophenotype-Based AQP3 and AQP5 Expression

AQP3 and AQP5 expression in tumor and stroma was analyzed in paired biopsy samples: pre-therapy core biopsies and matched post-therapy tumor bed tissue specimens (shown in Fig. 2). Baseline expression levels showed no significant differences between HER2+ breast cancer and TNBC. However, neoadjuvant chemotherapy induced significant increase in AQP3 expression in tumor cells across both immunophenotypes (HER2+ *n* = 75, mean difference [MD] = 16.36%, 95% confidence interval (CI) [4.26%, 28.45%], *p* = 0.006; TNBC *n* = 56, MD = 22.83%, 95% CI [11.69, 33.96], *p* < 0.001, shown in Fig. 2a). Intergroup analysis revealed no significant differences between the two cohorts posttreatment.

**Fig. 2.**
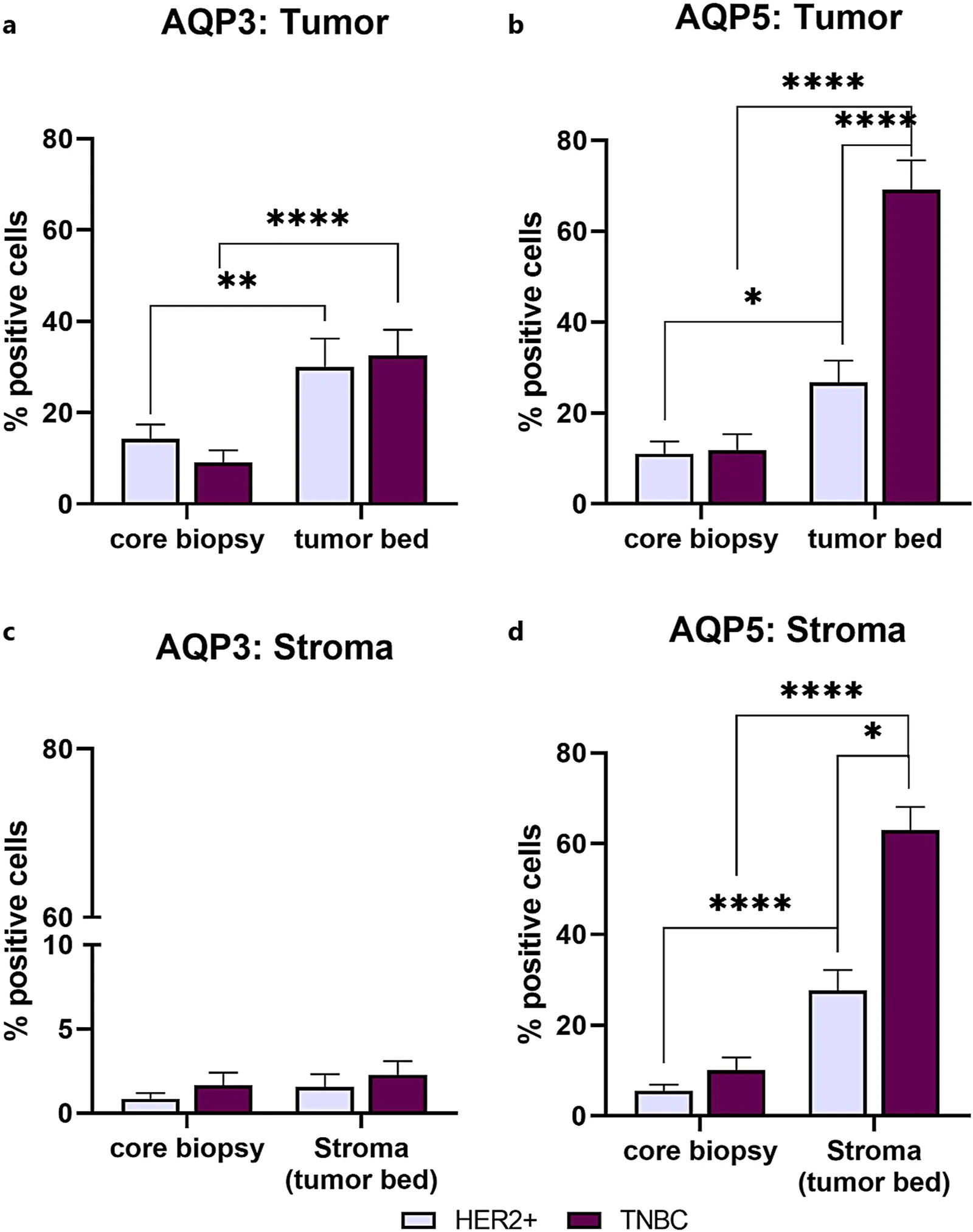
Levels of AQP3 (panels **a** and **c**) and AQP5 (panels **b** and **d**) in tumor (panels **a, b**) and stroma (panels **c**, **d**) in core biopsy and tumor bed tissue surgically removed according to immunophenotype HER2+ *n* = 75; TNBC *n* = 56. Results are presented as mean ± SEM; *p < 0.05; **p < 0.01; ***p < 0.001.

In contrast to AQP3, a significant difference in AQP5 expression was detected between HER2+ breast cancer and TNBC. Chemotherapy significantly increased AQP5 expression in both the tumor and the surrounding stroma. Specifically, AQP5 expression in tumor cells increased significantly in both immunophenotypes (HER2+: MD = 16.36%, 95% CI [4.26%, 28.45%], *p* = 0.029; TNBC: MD = 22.83%, 95% CI [11.69%, 33.96%], *p* = 0.010; Fig. 2b), as did stromal AQP5 expression (HER2+: MD = 22.10%, 95% CI [11.87%, 32.34%], *p* < 0.001; TNBC: MD = 52.95%, 95% CI [40.74%, 65.15%], *p* < 0.001; Fig. 2d). When comparing the two immunophenotypes after treatment, AQP5 expression was significantly more pronounced in TNBC than in HER2+ malignancies, demonstrating a consistent trend in both the tumor (MD = 42.81%, 95% CI [24.73%, 60.89%], *p* < 0.001, shown in Fig. 2b) and the stroma (MD = 35.95%, 95% CI [17.72%, 54.19%], *p* = 0.019, shown in Fig. 2d).

### pCR-Based AQP3 and AQP5 Expression

Next, AQP3 and AQP5 expression levels were evaluated in tumor and stromal tissues relative to therapeutic response (pCR vs. non-pCR) (shown in Fig. 3). In pretreatment core biopsies, AQP3 and AQP5 expression levels in tumor cells and stroma showed no significant differences between the pCR and non-pCR groups. However, chemotherapy induced a significant increase in both AQP3 and AQP5 expressions in tumor cells within the non-pCR cohort (non-pCR *n* = 83; AQP3: MD = 19.35%, 95% CI [10.44%, 28.26%], *p* < 0.001, shown in Fig. 3a; AQP5: MD = 46.37, 95% CI [35.20, 57.54], *p* < 0.001, shown in Fig. 3b). This longitudinal alternation could not be observed in the pCR group (*n* = 0 for residual invasive tumor; *n* = 4 retained residual DCIS) due to the complete pathological absence of residual invasive tumor cells. Yet, the stroma provided clear results. Stromal AQP3 expression levels, which were initially negligible (<5% positive cells), increased significantly after the chemotherapy in the non-pCR group (*n* = 83; MD = 1.61, 95% CI [0.19, 3.03], *p* = 0.023, shown in Fig. 3c). More importantly, AQP5 expression levels in the stroma of the non-pCR group significantly increased (*n* = 83; MD = 47.35%, 95% CI [38.55%, 56.14%], *p* < 0.001; Fig. 3d), whereas no change in stromal AQP5 expression level was observed in the pCR group.

**Fig. 3.**
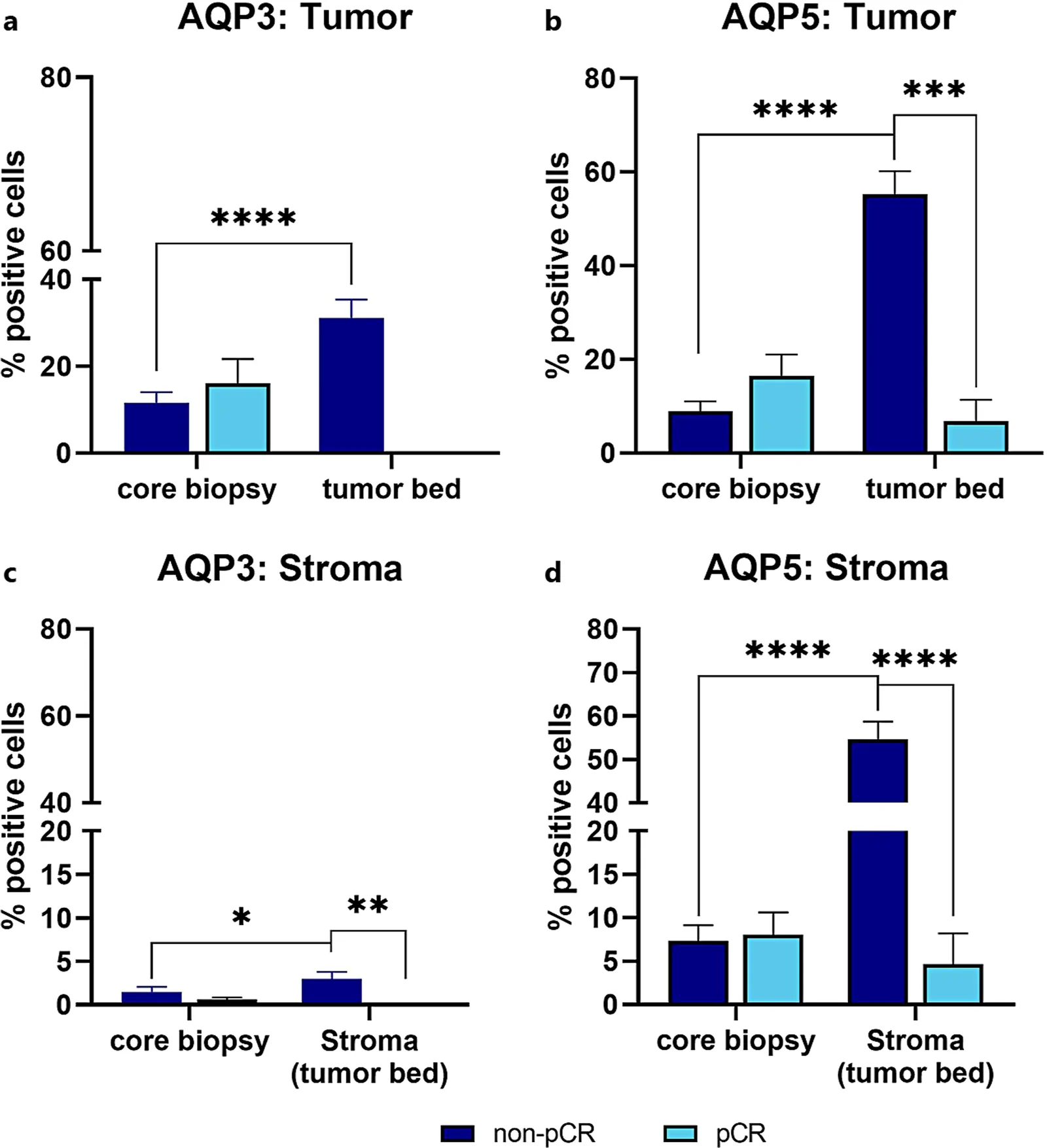
Levels of AQP3 (panels **a** and **c**) and AQP5 (panels **b** and **d**) in tumor (panels **a**, **b**) and stroma (panels **c**, **d**) in core biopsy and tumor bed tissue surgically removed after therapy, according to treatment response. Non-pCR tumor and stroma *n* = 83; pCR tumor *n* = 11 and stroma *n* = 48. Tumor cell analysis in the pCR group reflects residual DCIS only, as invasive tumor cells are absent by definition. Results are presented as mean ± SEM; *p < 0.05; **p < 0.01; ***p < 0.001.

### Immunophenotype-Based NRF2 Expression and Localization

To assess changes in the cellular antioxidative response, the intracellular expression and subcellular localization of NRF2 were analyzed (shown in Fig. 4). Interestingly, HER2+ breast cancer cells (*n* = 75) exhibited no significant change in nuclear NRF2 following therapy and even demonstrated a significant decrease in cytoplasmic NRF2 levels (MD = 18.18%, 95% CI [1.03%, 35.34%], *p* < 0.001; shown in Fig. 4a). Conversely, TNBC cells (*n* = 56) exhibited a significant increase in nuclear NRF2 following the therapy (MD = 22.79%, 95% CI [4.12%, 41.46%], *p* = 0.007). Although there was no longitudinal alteration in cytoplasmic NRF2 in TNBC, cytoplasmic levels of NRF2 were consistently higher in TNBC compared to HER2+ breast cancer (MD = 26.46%, 95% CI [7.40%, 45.53%], *p* = 0.001). In the stroma, nuclear NRF2 significantly increased following therapy in both immunophenotypes (HER2+ MD = 13.15%, 95% CI [2.58%, 23.72%], *p* = 0.006; TNBC MD = 26.94%, 95% CI [12.94%, 40.94%], *p* < 0.001 shown in Fig. 4b). However, TNBC stroma exhibited significantly higher nuclear NRF2 levels than HER2+ breast cancer stroma following neoadjuvant chemotherapy (MD = 8.07%, 95% CI [0.66%, 15.47%] *p* = 0.005). Additionally, TNBC demonstrated an increased cytoplasmic NRF2 expression after the chemotherapy (MD = 17.41%, 95% CI [5.27%, 29.55%], *p* = 0.001), a feature not observed in HER2+ breast cancer.

**Fig. 4.**
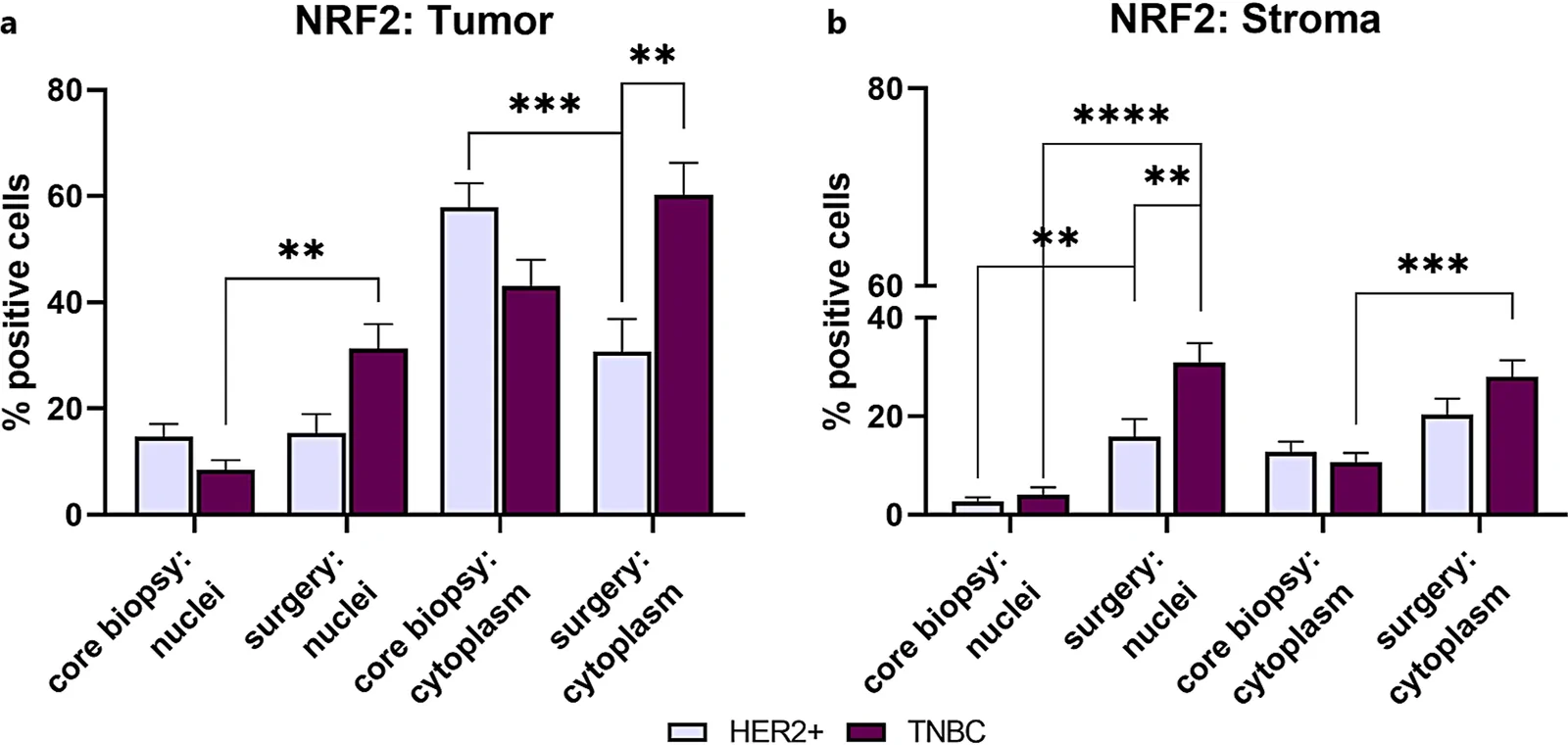
NRF2 levels in tumor (panel **a**) and stroma (panel **b**) in core biopsy and tumor bed tissue surgically removed according to immunophenotype HER2+ *n* = 75; TNBC *n* = 56. Results are presented as mean ± SEM; **p < 0.01; ***p < 0.001; ****p < 0.0001.

### pCR-Based NRF2 Expression and Localization

NRF2 intracellular expression and localization were next stratified according to pathological response (pCR vs. non-pCR) (shown in Fig. 5). A significant increase in nuclear NRF2 following therapy was detected only in the non-pCR group (*n* = 83; MD = 17.51%, 95% CI [1.23%, 33.79%], *p* = 0.024 for tumor cells; MD = 27.28%, 95% CI [15.37%, 39.19%], *p* < 0.001 for stroma). Interestingly, residual DCIS tumor cells detected in the pCR group exhibited significantly lower NRF2 expression in both the nucleus (MD = 17.06%, 95% CI [1.52%, 32.60%], *p* = 0.019) and cytoplasm (MD = 35.86%, 95% CI [20.32%, 51.40%], *p* < 0.001) compared to the non-pCR group. Similarly, nuclear NRF2 was significantly lower following therapy in the pCR group compared to the non-pCR group (MD = 18.34%, 95% CI [4.88%, 31.80%], *p* = 0.003). Cytoplasmic positivity was also increased in stroma in the non-pCR group after the chemotherapy (MD = 16.03%, 95% CI [4.12%, 27.94%], *p* = 0.004), indicating a potential expansion of the total cellular NRF2 pool associated with therapeutic response.

**Fig. 5.**
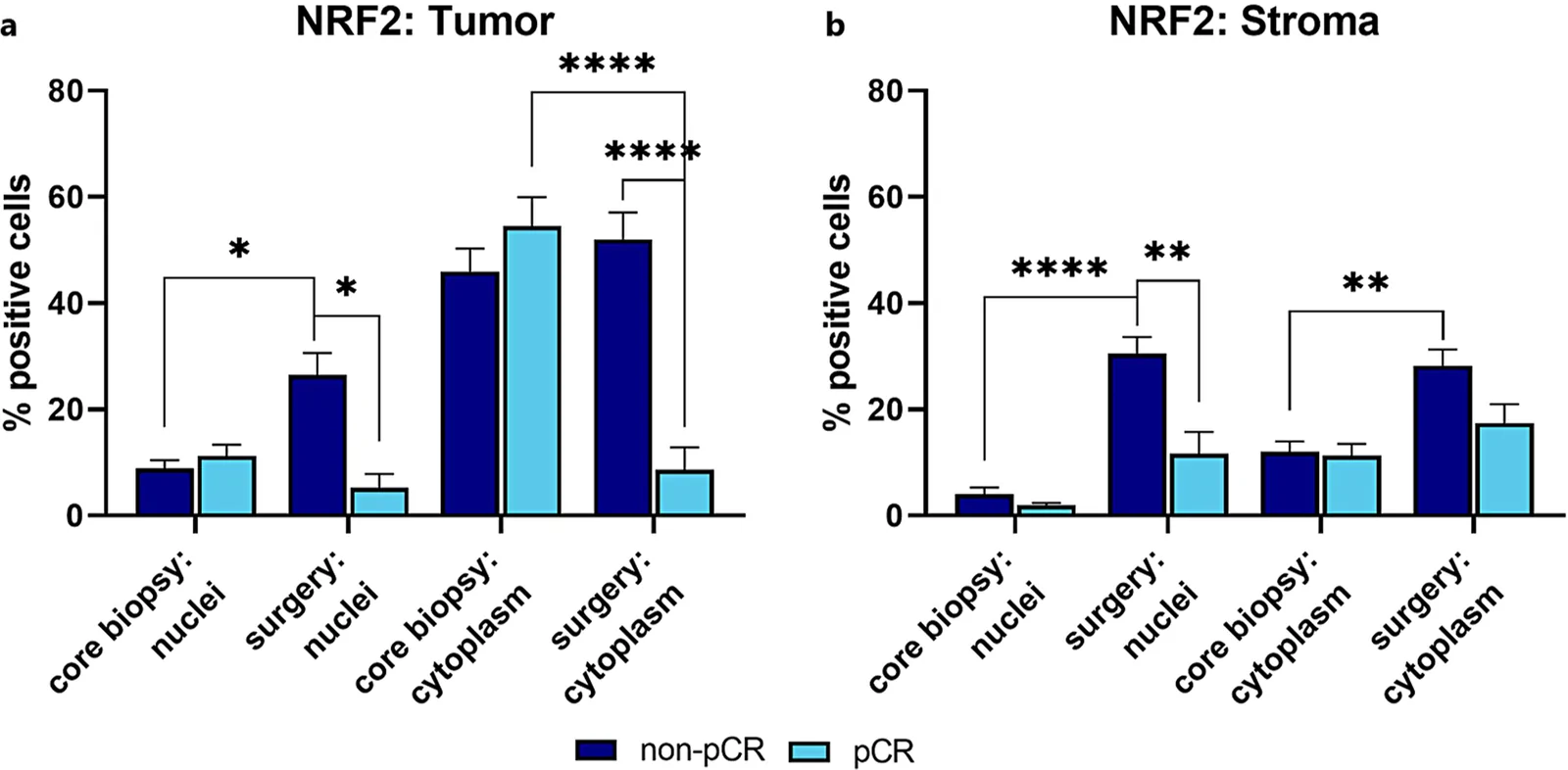
NRF2 levels in tumor (panel **a**) and stroma (panel **b**) in core biopsy and tumor bed tissue surgically removed after therapy according to treatment response. Non-pCR tumor and stroma *n* = 83; pCR tumor *n* = 11 and stroma *n* = 48. Tumor cell analysis in the pCR group reflects residual DCIS only, as invasive tumor cells are absent by definition. Results are presented as mean ± SEM; *p < 0.05; **p < 0.01; ****p < 0.0001.

### Correlation Analyses

To establish correlations between the measured parameters and therapy response, parameters were analyzed using Spearman’s correlations (only statistically significant correlations are described; complete data have been presented in Table 2.). The analysis confirmed a very strong correlation between residual cancer burden (RCB) and complete therapy response in HER2+ breast cancer and a moderate correlation in TNBC, thereby confirming the robustness of our analysis.

**Table 2.** Spearman’s correlation analyses

​		HER2+		TNBC	
		Spearman’s rho	*p* value	Spearman’ rho	*p* value
RCB	pCR	−0.901	<0.001	−0.632	<0.001
	AQP5 in tumor cells-tumor bed	0.642	<0.001	0.413	0.003
	AQP3 in stroma-tumor bed	NS	NS	0.454	0.001
	NRF2 in tumor cell cytoplasm-core biopsy	NS	NS	−0.387	0.009
	NRF2 in stroma nuclei-tumor bed	NS	NS	0.510	<0.001
	NRF2 in stroma cytoplasm-tumor bed	NS	NS	0.319	0.035
pCR	AQP5 in tumor cells-tumor bed	−0.500	<0.001	−0.636	<0.001
	AQP5 in stroma-tumor bed	0.303	0.010	​	​
	AQP3 in stroma-tumor bed	NS	NS	0.352	0.011
	NRF2 in stroma nuclei-tumor bed	NS	NS	0.397	0.006
	NRF2 in stroma cytoplasm-tumor bed	NS	NS	0.589	<0.001
AQP5 in tumor cells-core biopsy	AQP5 in stroma-core biopsy	0.811	<0.001	0.877	<0.001
	AQP3 in tumor cells-core biopsy	0.264	0.031	NS	NS
	NRF2 in tumor cell nuclei-core biopsy	0.412	<0.001	NS	NS
	NRF2 in stroma nuclei-core biopsy	0.313	0.012	NS	NS
	NRF2 in stroma cytoplasm-core biopsy	0.269	0.032	NS	NS
AQP5 in tumor cells-tumor bed	NRF2 in stroma nuclei-tumor bed	0.353	0.013	0.367	0.011
	NRF2 in stroma cytoplasm-tumor bed	NS	NS	0.383	0.008
AQP3 in tumor cells-core biopsy	AQP3 in stroma-core biopsy	NS	NS	0.503	<0.001
	AQP3 in tumor cells-tumor bed	NS	NS	0.448	0.005
AQP3 in stroma-core biopsy	AQP3 in tumor cells-core biopsy	0.454	<0.001	NS	NS
	NRF2 in stroma nuclei-tumor bed	−0.315	0.033	NS	NS
	Ki67	​	​	−0.351	0.010
AQP3 in tumor cells-tumor bed	NRF2 in tumor cell nuclei-core biopsy	NS	NS	−0.368	0.027
NRF2 in tumor nuclei-core biopsy	NRF2 in tumor cell cytoplasm-core biopsy	0.516	<0.001	0.764	<0.001
	NRF2 in stroma nuclei-core biopsy	0.398	0.001	0.544	<0.001
	NRF2 in stroma cytoplasm-core biopsy	0.382	0.002	0.594	<0.001
	AQP5 in stroma-core biopsy	0.318	0.012	NS	NS
NRF2 in tumor cell cytoplasm-core biopsy	NRF2 in stroma nuclei-core biopsy	0.347	0.006	0.440	0.002
	NRF2 in stroma cytoplasm-core biopsy	0.371	0.003	0.485	<0.001
NRF2 in tumor cell nuclei-core biopsy	NRF2 in tumor cell cytoplasm-core biopsy	NS	NS	0.764	<0.001
	NRF2 in stroma nuclei-core biopsy	NS	NS	0.544	<0.001
	NRF2 in stroma cytoplasm-core biopsy	NS	NS	0.594	<0.001
NRF2 in stroma nuclei-core biopsy	NRF2 in stroma cytoplasm-core biopsy	0.589	<0.001	0.663	<0.001
	NRF2 in tumor cell cytoplasm-tumor bed	0.591	0.016	​	​
NRF2 in tumor cell nuclei-tumor bed	NRF2 in tumor cell cytoplasm-tumor bed	NS	NS	0.489	0.003
NRF2 in tumor cell cytoplasm-tumor bed	NRF2 in stroma cytoplasm-tumor bed	NS	NS	0.359	0.034
NRF2 in stroma cytoplasm-core biopsy	AQP5 in stroma-core biopsy	0.297	0.017	NS	NS
	Ki67	0.386	0.003	NS	NS
NRF2 in stroma nuclei-tumor bed	NRF2 in stroma cytoplasm-tumor bed	0.604	<0.001	0.624	<0.001
	AQP5 in stroma-core biopsy	−0.294	0.040	​	​

Only significant correlations are presented in the table.

In HER2+ breast cancer, RCB showed moderate correlation with AQP5 in tumor cells after chemotherapy (Spearman’s *ρ* = 0.642) and moderate negative correlation with pCR (Spearman’s *ρ* = −0.500). Interestingly, pCR moderately correlated with AQP3 in stroma (Spearman’s *ρ* = 0.303). Furthermore, there was a strong correlation between AQP5 in the tumor and stroma within pretreatment core biopsies (Spearman’s *ρ* = 0.811), and a moderate correlation with NRF2 in nuclei of tumor and stromal cells. Following AQP5, AQP3 was moderately correlated between the tumor and stroma in core biopsies (Spearman’s *ρ* = 0.454). In TNBC, RCB moderately correlated with AQP3 in tumor cells and AQP3 in the tumor stroma (Spearman’s ρ = 0.413 and ρ = 0.454, respectively) and with NRF2 in the nuclei and cytoplasm of stromal cells after the therapy (Spearman’s *ρ* = 0.510 and *ρ* = 0.319, respectively). Pre-therapy cytoplasmic NRF2 levels in core biopsy tumor cells moderately correlated negatively with the RCB (Spearman’s *ρ* = −0.387). Furthermore, pCR was moderately negatively correlated with postsurgical tumor AQP5 expression (Spearman’s *ρ* = −0.636), stromal AQP3 expression (Spearman’s *ρ* = −0.352$), and stromal nuclear NRF2 expression (Spearman’s *ρ* = −0.397), as well as with stromal cytoplasmic NRF2 expression (Spearman’s *ρ* = −0.589). There was a strong positive correlation in the AQP5 expression between the tumor and stroma in the core biopsies (Spearman’s *ρ* = 0.877). After therapy, AQP5 also moderately correlated with NRF2 expression in the nuclei and cytoplasm of the stroma (Spearman’s *ρ* = 0.367 and *ρ* = 0.383, respectively).

## Discussion

Breast cancer remains one of the leading causes of death among women [19]. Successful treatment is still challenged by the development of tumor resistance and associated side effects, including oxidative stress. Consequently, identifying predictive markers of therapy response is crucial. Chemotherapy forms the therapeutic backbone for both TNBC and HER2+ breast cancer. This strategy is essential for TNBC due to its lack of specific molecular targets, and for HER2+ breast cancer because the pathological diagnosis only requires 10% HER2+ tumor cells, often leaving a great portion of tumor cells unaffected by HER2-targeted therapy. Chemotherapy follows the standard AC neoadjuvant regimen in 4–6 cycles, followed by 12 cycles of weekly paclitaxel. In TNBC patients with *BRCA1/2* mutations, olaparib could also be used in the neoadjuvant setting, and in patients with high-risk TNBC, a combination of pembrolizumab, carboplatin, and paclitaxel can be used in addition to the aforementioned cytostatics [20]. All patients in this study received 4 to 6 AC cycles and 12 weeks of paclitaxel, as our samples were obtained before the introduction of olaparib, pembrolizumab, and carboplatin into the neoadjuvant protocol. According to the clinical guidelines, our patients were treated with protocol that provided us with paired core biopsy samples (before therapy) and resected tumor bed samples (after therapy), allowing us to follow the dynamics of AQP3, AQP5, and NRF2 expression following treatment and link them to the pCR.

To date, most clinical studies have focused on AQP profiling at a single sampling point, establishing that aberrant AQP3 and AQP5 overexpression strongly correlates with aggressive breast cancer phenotypes and can serve as reliable prognostic indicators [8, 21]. In contrast, our study evaluated the dynamic pre- and posttreatment changes of these AQPs. We initially hypothesized that distinct differences in AQP3 and AQP5 levels would exist between subtypes before therapy; however, our analysis revealed that basal expression levels were remarkably similar across both the tumor and surrounding stroma in both TNBC and HER2-positive breast cancers. Notably, posttreatment samples exhibited a significant increase in both AQP3 and AQP5 expressions in the tumor and stroma compared to pretreatment biopsies. Furthermore, TNBC demonstrated a significantly higher increase in AQP5 in the tumor and stroma after chemotherapy compared to HER2+ breast cancer, which is in line with previous studies linking AQP5 to more malignant types of breast cancer [7, 8].

When patients were regrouped according to pCR, higher AQP5 levels were observed after the chemotherapy in the non-pCR group in both tumor cells and stroma. Conversely, AQP5 levels remained unchanged in the stroma of the pCR group. These findings suggest that the accumulation or persistence of AQP5 in the stroma following chemotherapy may be associated with a mechanism of therapy failure. This is a novel finding in clinical samples, as current literature lacks data on AQP expression patterns before and after chemotherapy, especially within the tumor stroma. AQP expression has been studied in skin cancer biopsies, but without addressing changes due to therapy, where AQP3 was unchanged while AQP1 increased [22]. The biological context is supported by in vitro data demonstrating that silencing AQP5 in MCF7 cells reverses Adriamycin resistance, decreases P-glycoprotein and glutathione S-transferase π levels, and activates apoptosis [23]. The clinical data on AQPs remain inconclusive. While one study associated hormone-positive and HER2+ early-stage breast cancer and AQP5 with better survival [24], our data indicated that under the selective pressure of neoadjuvant chemotherapy, increases in AQP5 in the tumor and stroma are linked to an incomplete response to the therapy.

Given that AQP3 and AQP5 are peroxiporins, and oxidative stress is associated with conventional chemotherapy, but also with radiotherapy and immunotherapy [25], we evaluated the antioxidant transcription factor NRF2 into consideration as a potential effector pathway. Our data confirm that chemotherapy exposure is associated with the activation of NRF2, probably in response to oxidative stress, but exhibits a cell-type and immunotype-specific pattern. NRF2 levels increased significantly in TNBC cell nuclei after the chemotherapy, but showed no significant change in HER2+ breast cancer cells. These results are in line with an in vitro study which demonstrated that inhibition of NRF2 in breast cancer stem cells sensitized the cells to radiation [26]. Interestingly, NRF2 increased in the nuclei of the stroma of both TNBC and HER2+ breast cancer, suggesting a cell-type-specific response. Further, nuclear NRF2 after chemotherapy correlated with incomplete therapy response (non-pCR). This pattern was even more pronounced in the stroma, where nuclear NRF2 significantly increased only in the non-pCR group. These findings are consistent with a study linking high NRF2 activation (p-NRF2) in TNBC to poorer prognosis [27], although this study lacked post-therapy data. Moreover, in lung cancer, the p62-NRF2 pathway regulates the transition of normal fibroblast in the tumor microenvironment to cancer-associated fibroblast phenotype which supports cancer growth [28]. However, a study analyzing *NFE2L2* gene expression in breast cancer core biopsies correlated NRF2 with immune checkpoint proteins in ER-positive/HER2-negative breast cancer rather than with pCR [29], indicating complex regulation between gene and protein expression and protein activation.

To establish the clinical relevance of our findings, we evaluated the expression of our target proteins against therapy response indicators. Across both subtypes, pre-therapy samples revealed synchronization of AQP5 between the tumor and stroma, which co-localized with nuclear NRF2. Mechanistically, this link is plausible, AQP5 functions as a peroxiporin, regulating the H_2_O_2_ flux in and out of the cells, which can further activate the NRF2 pathway bolstering oxidative stress defense. Following neoadjuvant chemotherapy, distinct resistance patterns occur. In TNBC, post-therapy AQP3 expression in both the tumor and stroma correlated with higher RCB suggesting the role in therapy resistance. Similarly, poor pCR correlated with AQP5 in the tumor after therapy, AQP3 in stroma, and NRF2 in the nuclei of the stroma. The correlation underlines a relationship between AQP5 and NRF2 expression in stroma. Together, these correlation patterns point to possible connection between AQP5 and NRF2 in the stroma after neoadjuvant chemotherapy, possibly acting as a survival mechanism in nonresponsive tumors.

Several limitations linked to our retrospective, correlative study must be noted. First, comparing the pre-therapy core biopsies and post-therapy samples provides a span in a time frame for cancer adaptation. Thus, the observed shifts in our target proteins can be the result of the clonal expansion of preexisting resistant subpopulation, rather than direct induction. Next, while we controlled for structural variations by evaluating positivity within the tumor and stroma independently, neoadjuvant treatment changes the tumor-to-stroma ratio. These shifts across the time points of sampling mean that microenvironmental remodeling itself could influence the observed expression patterns, a factor that remains an inherent challenge in longitudinal tissue-based studies.

Combining AQP3 and AQP5 with NRF2 presents a potential strategy for overcoming therapeutic resistance. While these proteins play a role in tumor survival, further research is needed to distinguish the exact mechanisms of their upregulation. Specifically, we need to determine if elevated expression in the tumor and stroma is a direct, chemotherapy-induced adaptation, or if a small, resistant fraction of cells possesses an inherent selective advantage. Additionally, the precise role of the stromal AQP expression must be clarified to establish whether AQP can actively support a pro-tumor niche.

## Conclusion

Our findings demonstrate that AQP5 upregulation and NRF2 nuclear localization in both the tumor and stroma are significantly associated with an incomplete pathological response (non-pCR) after neoadjuvant therapy in HER2+ and TNBC. These results suggest that the tumor microenvironment may participate in an adaptive response to treatment, pointing to AQP5 and NRF2 as potential candidates in the development of therapeutic resistance. Consequently, AQP5 and NRF2 need further validation as potential therapeutic targets or indicators of conventional therapy resistance.

## Statement of Ethics

This study was performed in line with the principles of the Declaration of Helsinki. Approval was granted by the Ethics Committee of the University Hospital Centre “Sestre milosrdnice” issued on the October 6, 2022, under Accession No. 251-29-11-21-01-10. Due to retrospective character of the study and use of paraffin-embedded tissue, written informed consent was not required.

## Conflict of Interest Statement

The authors have no conflicts of interest to declare.

## Funding Sources

This work was supported by Ruđer Bošković Institute, Zagreb, Croatia.

## Author Contributions

Ilija Guteša, Sanda Bubanović, and Ana Čipak Gašparović contributed to the study conception and design. Material preparation, data collection, and analysis were performed by all authors. The first draft of the manuscript was written by Ilija Guteša and Ana Čipak Gašparović, and all authors commented on previous versions of the manuscript and read and approved the final manuscript.

## Data Availability

The datasets generated and/or analyzed during the current study are available from the corresponding author on reasonable request.
